# Tackling adversity through innovation: A pilot study exploring VR as a tool to identify and diagnose depression

**DOI:** 10.1192/j.eurpsy.2024.77

**Published:** 2024-08-27

**Authors:** S. Sutori, Karolinska Institutet: E. Eliasson, G. Hadlaczky, I. Todorov, V. Carli; University of Padua: F. Mura, V. Cardi; Polytechnic University of Valencia: V. Ortiz, M.A. Raya; University of Pisa: A.L. Alfeo, V. Catrambone, G. Valenza

**Affiliations:** ^1^National Centre for Suicide Research and Prevention (NASP), LIME, Karolinska Institutet, Solna, Sweden

## Abstract

**Introduction:**

The final aim of the EXPERIENCE project is to enable individuals to record and share extended-personal realities in Virtual Reality (VR) - which entails the consideration of a person’s neurophysiological, psychological, and cognitive states. One prospective application is using this technology to aid in assessing symptoms of affective disorders.

**Objectives:**

The objective is to test the ability of a pre-designed VR environment to differentiate between individuals with depressive symptoms and healthy controls (HCs) via machine learning algorithms.

**Methods:**

Conducted as a pilot study in Italy, we recruited 100 volunteers, comprising 50 HCs and 50 individuals with moderate depressive symptoms assessed via the PHQ-9. Through a 40–60-minute VR engagement, comprehensive data on cognitive (inc. cognitive flexibility, sustained attention, working memory, processing speed), behavioral (exploration, attentional bias), and physiological (heart-rate variability, skin conductance) variables was collected. Subsequently, an explainable artificial intelligence model (xAI) was trained on data from 80% of the sample and tested on the remaining 20% in terms of accuracy for between-group classification.

**Results:**

Following an iterative process that considered both the importance assigned to each variable in the different models and the theoretical relevance of these variables to depression the final model achieved an average accuracy of 71% (with individual trials ranging from 64.5% to 77.1%). Key predictors included exploratory behaviors and heart-rate variability during both exploration and cognitive tasks.

**Conclusions:**

These results are comparable, however remain below the levels of accuracy achieved based on fMRI and DTI data alone (around 80%). Nonetheless, the EXPERIENCE system, slated for refinement beyond this pilot phase, shows potential in integrating multimodal data for evaluating affective disorder symptoms, aiming for a more objective screening and diagnostic approach at a lower cost.

**Acknowledgement:**

The EXPERIENCE project is funded by the European Commission H2020 Framework Program, Grant No. 101017727.

**Disclosure of Interest:**

None Declared

